# Treatment outcomes of unknown primary squamous cell carcinoma of the head and neck

**DOI:** 10.1371/journal.pone.0205365

**Published:** 2018-10-18

**Authors:** Yu-Hsuan Hung, Shih-An Liu, Chen-Chi Wang, Ching-Ping Wang, Rong-San Jiang, Shang-Heng Wu

**Affiliations:** 1 Department of Otolaryngology–Head and Neck Surgery, Taichung Veterans General Hospital, Taichung, Taiwan; 2 Department of Otolaryngology, School of Medicine, National Yang-Ming University, Taipei, Taiwan; 3 School of Speech Language Pathology and Audiology, Chung Shan Medical University, Taichung, Taiwan; 4 School of Medicine, Chung Shan Medical University, Taichung, Taiwan; Duke Cancer Institute, UNITED STATES

## Abstract

**Background:**

Treatment modality of unknown primary squamous cell carcinoma of the head and neck (SCCHN) remains controversial.

**Objectives:**

To evaluate the treatment outcomes and prognostic factors of unknown primary SCCHN.

**Materials and methods:**

Patients with unknown primary SCCHN from April 1995 to March 2013 were recruited retrospectively.

**Results:**

Sixty-nine patients were enrolled. The median time of follow-up was 55.5 months. The 2-year loco-regional control rate of all the patients was 60.4%. Multivariate Cox regression analysis revealed that N3 stage, extracapsular spread, distant metastasis, and treatment modality were significantly associated with neck recurrence. The actuarial 5-year disease-specific survival rates of neck dissection, neck dissection plus adjuvant therapy, radiotherapy alone, and combined therapy were 80.0%, 61.7%, 33.3%, and 68.8%, respectively (p = 0.046). The 5-year disease-specific survival rates of N1/N2a, N2b/N2c, and N3 stage were 83.9%, 64.3%, and 36.7%, respectively (p = 0.013). Univariate regression analysis revealed that neck recurrence, supraclavicular node involvement, distant metastasis, N3 stage, and unhealthy lifestyle habits were correlated with disease-specific mortality, especially the first three parameters. Patient’s occupation and comorbidity were not significantly correlated with survival.

**Conclusions:**

Composite therapy is mandatory for advanced unknown primary SCCHN. Supraclavicular node involvement and unhealthy lifestyle habits, such as betel nut chewing, indicate a poor prognosis.

## Introduction

Cervical adenopathy is a well-known initial manifestation of head and neck cancer. In the histopathologies of head and neck cancers, over 90% were diagnosed as squamous cell carcinoma [1]. In most cases, the primary origin of cancer could be discovered during a thorough head and neck examination, along with various imaging studies. In one to nine percent of these patients, the primary tumor had yet to be determined, even after an aggressive evaluation [2–4]. Several treatment modalities including surgery, radiotherapy, chemotherapy, molecular targeted therapy, or a combination of these have been proposed [3–6]. Owing to the low incidence of unknown primary cancers and the fact that only a few randomized clinical trials have been conducted, the biology, optimal diagnostic algorithm, and treatment strategy remain key challenges.

Alcohol consumption, betel nut chewing, and smoking have been identified as major risk factors for head and neck cancer in many parts of Asia [7,8]. However, there are few data in the literature regarding the effects of these unhealthy lifestyle habits, patient’s occupation, and comorbidity rates on the survival of patients with unknown primary squamous cell carcinoma of the head and neck (SCCHN) in areca quid chewing areas. Therefore, the purpose of the present study was to evaluate both the treatment outcomes and prognostic factors of unknown primary SCCHN.

## Material and methods

### Patient population

We retrospectively reviewed patients with unknown primary cancer of the head and neck who were diagnosed at Taichung Veterans General Hospital between April 1995 and March 2013. A thorough review of each patient’s medical charts was conducted. The main exclusion criteria were histopathology with evidence of non-squamous cell carcinoma, incomplete or without therapy, and loss of follow-up. This study was approved by the Institutional Review Board of Taichung Veterans General Hospital.

### Diagnostic workup

The N stage for all patients was classified retrospectively according to the American Joint Committee for Cancer Staging (AJCC) 2010 classification. Extracapsular spread (ECS) was defined either by pathological proof or by image findings showing the presence of indistinct nodal margins, irregular nodal capsular enhancement, or infiltration into the adjacent fat or muscle. The initial diagnosis was made either by a fine-needle aspiration (FNA), or an excisional/incisional biopsy. All of the patients received a plan film chest X-ray, head and neck computed tomography (CT) scan or magnetic resonance image (MRI), whole-body bone scan, and abdominal sonography. The use of 18F-fluorodeoxyglucose positron emission tomography (18FDG-PET) scan was limited to twenty-one of the patients due to the high cost of this imaging modality, which has also only been available in our institute since 2002. Each patient also received a panendoscopy with random biopsy under general anesthesia, and this included nasopharyngoscopy, laryngoscopy, and esophagoscopy. The sites of biopsy were the nasopharynx, base of tongue, and hypopharynx. Bilateral tonsillectomies were also performed routinely as part of the diagnostic work-up. In addition, multidisciplinary consultations including chest, gastro-intestinal, colorectal, genito-urinary, and gynemetrics were performed.

### Treatment modality

Treatment modalities were classified into neck dissection (ND) alone, ND with adjuvant therapy, radiotherapy (RT) alone, and combined therapy. Adjuvant therapy consisting of RT alone, chemotherapy with RT and concurrent chemoradiation began three to five weeks after surgery. Combined therapy was composed of chemotherapy plus radiotherapy and concurrent chemoradiation. The diverse treatment modalities were prescribed depending upon the nodal stage, the performance status, and willingness of patients.

The neck dissections consisted of a comprehensive radical dissection or modified dissection, which were performed either unilaterally or bilaterally according to the CT scan or MRI findings.

Patients received standard fractionated radiotherapy of 2 Gy per fraction, at a standard of five fractions per week. The radiation doses of definite and adjuvant radiotherapy were 66 to 72 Gy and 60 to 66 Gy, respectively. The field of radiation included the potential mucosal primaries such as the nasopharynx, base of tongue, tonsillar fossa, and hypopharynx, along with the bilateral neck, while sparing the larynx.

The chemotherapy regimens were mainly cisplatin at 100 mg/m2, and 5-fluorouracil(5-FU) at 400 mg/m2. Chemoradiation was conducted with high-dose cisplatin at 100 mg/m2 once per three weeks during the period of radiotherapy.

### Follow-up

All the patients were visited regularly on a monthly basis in year 1, bimonthly in year 2, and quarterly in years 3 to 5 at the multidisciplinary head and neck clinic.

### Endpoints and statistics

The primary outcome of this study was regional recurrence, while the secondary outcome was disease-specific survival (DSS). All of these time-to-event variables were measured from the date of therapy. Loco-regional control (LRC) was defined as neck nodal control from the day of treatment to the day of loco-regional failure, which was proved by histopathology. For the patients with a persistent tumor of the neck which did not regress, the date of regional failure was defined as the date of completion of therapy. DSS was measured from the date of treatment to the last follow-up visit or death, according to the Kaplan-Meier estimator. Continuous variables were presented as the mean standard deviation. The significance of difference in survival between the groups was calculated by the log rank test. The Cox regression model was used to calculate the prognostic factors for recurrence and survival, adjusting for uni- and multi-covariates. The difference of categorical data between the two groups was calculated by the t test, Chi-Square test, and Fisher’s exact test. A p value < 0.05 was considered statistically significant. IBM SPSS 22.0 for Windows was used for data management and statistical analysis.

## Results

### Patient characteristics

A total of 69 patients with unknown primary SCCHN were eligible for recruitment. The mean age of the patients was 55.7 years (range, 34–78 years), including 57 male and 12 females. The number of patients with a history of alcohol consumption, areca quid chewing, and smoking was 34 (49.3%), 28 (40.6%), and 46 (66.7%), respectively. There were 12 cases (17.4%) with comorbid diabetic mellitus and 16 cases (23.2%) with hypertension. Fifty-five patients (79.7%) received a FNA, while the others were diagnosed by excisional or incisional biopsy, due to an insufficient FNA results. The most common histopathology was poorly differentiated carcinoma (41, 59.4%), followed by moderately differentiated carcinoma (21, 30.4%) and undifferentiated carcinoma (7, 10.1%). The numbers of cases classified as N1, N2a, N2b, N2c and N3 were 1, 12, 32, 4, and 20, respectively. As there was only one N1 case and almost none of the N2a cases had ECS, we adjusted the nodal stage to N1/N2a, N2b/N2c, and N3. The laterality of the involved necks included 27 (39.1%) right side cases, 36 (52.2%) left side cases, and 6 (8.7%) bilateral cases. In order, the neck levels with most involvement were level II (55, 34.6%), III (41, 25.8%), IV (30, 18.9%), I (24, 15.1%), and V (9, 5.7%). There were 8 cases (11.6%) with supraclavicular lymph nodes involvement, 6 over the left side, and 2 cases bilaterally.

Five (7.2%) patients received ND alone, 40 (58.0%) had ND with adjuvant therapy, 9 (13.0%) patients received RT alone, and 15 (21.7%) had combined therapy. A total of 41 (59.4%) cases were diagnosed with ECS. The baseline demographic and clinical data are listed in Table 1.

**Table 1:** Demographic and clinical characteristics of patients.

	n	%
Age (mean ± SD)	55.7 ± 10.5	
Gender		
Male	57	82.6
Female	12	17.4
Occupation		
Blue collar	24	34.8
White collar	27	39.1
None	18	26.1
Alcohol	34	49.3
Betel nut	28	40.6
Smoking	46	66.7
Diabetic mellitus	12	17.4
Hypertension	16	23.2
Laterality		
L	36	52.2
R	27	39.1
Bil	6	8.7
Nodal stage		
N1/N2a	13	18.8
N2b/N2c	36	52.2
N3	20	29.0
Supraclavicular node	8	11.6
Extracapsular spread	41	59.4
Pathology^1^		
MD	21	30.4
PD	41	59.4
Undifferentiated	7	10.1
Treatment^2^		
ND	5	7.2
ND + adjuvant therapy	40	58.0
RT alone	9	13.0
Combined therapy	15	21.7
Appearance of primary	10	14.5

^1^Pathology: MD (moderate differentiated), PD (poorly differentiated)

^2^Treatment: ND (neck dissection), RT (radiation), combined therapy (chemotherapy + RT, concurrent chemoradiation)

### Disease-related characteristics

#### Locoregional failure

With a median follow-up of 55.5 months (range, 6–234 months), 29 patients experienced loco-regional recurrence or persistent tumor. Most of these cases (25/29) were classified as the advanced stage (N2b, N2c, and N3). The average time to loco-regional failure was 9.8 months (range, 3–52 months). Among these patients, 2 cases had neck recurrence which evolved over 24 months. All these patients accepted salvage surgery with adjuvant therapy or concurrent chemoradiation subsequently for the neck recurrence.

During the follow-up period, eight patients developed upper aerodigestive tract (UADT) cancer and two patients were diagnosed with lung cancer. The lesions in the UADT included three cases occurring in the oral cavity, two cases in the esophagus, two cases in the nasopharynx, and one case in the base of tongue. The average time to primary cancer emergence was 20.6 months (range, 2–50 months). These 10 cases also received radical intent treatment for the emerging primary tumor.

#### Distant failure

A total of 19 cases experienced distant metastasis. The sites of distant metastasis included bone, liver, lung, chest wall, pleura, mediastinum, and brain. The average time to detection of distant metastasis was 12.1 months (range, 2–44 months). Once the distant metastasis occurred, the median survival time for these patients was 6.1 months (range, 1–20 months). All these patients received palliative therapy afterwards.

#### Loco-regional control and disease-specific survival rate

**Fig 1:**
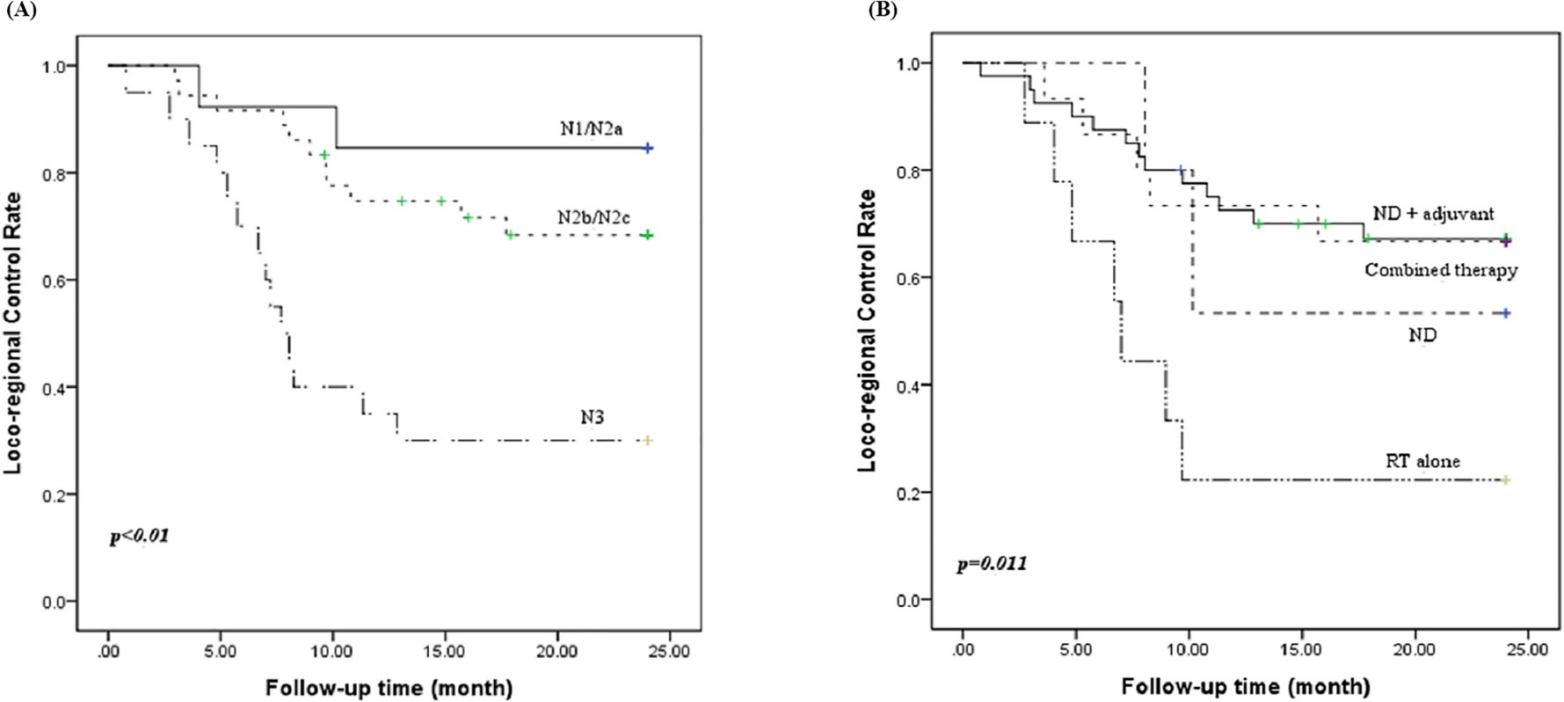
Loco-regional control rate according to N stage (A) and treatment modalities (B).

The 2-year actuarial LRC rate of all these patients was 60.4%. The 2-year actuarial LRC rate of N1/N2a, N2b/N2c, and N3 patients was 84.6%, 68.3%, and 30.0%, respectively (p <0.01) "Fig 1A". The 2-year actuarial LRC rates of ND, ND plus adjuvant therapy, RT alone, and combined therapy were 53.3%, 67.2%, 22.2%, and 66.7%, respectively (p = 0.011) "Fig 1B". The differences in individual categorical variables among patients with neck recurrence are shown in Table 2.

**Table 2:** Comparison between patients with or without neck recurrence.

	No (n = 42)		Yes (n = 27)		*p* value
	n	%	n	%	
Age (mean ± SD)	56.2 ± 10.8		54.9 ± 10.2		0.605
Gender^f^					0.518
Male	36	85.7	21	77.8	
Female	6	14.3	6	22.2	
Occupation					0.865
Blue	15	35.7	9	33.3	
White	17	40.5	10	37.0	
None	10	23.8	8	29.6	
Alcohol	19	45.2	15	55.6	0.403
Betel nut	15	35.7	13	48.1	0.305
Smoking	25	59.5	21	77.8	0.116
Diabetic mellitus^f^	5	11.9	7	25.9	0.194
Hypertension	12	28.6	4	14.8	0.186
Laterality					0.196
L	19	45.2	17	63.0	
R	20	47.6	7	25.9	
Bil	3	7.1	3	11.1	
Nodal stage					<0.01**
N1/N2a	11	26.2	2	7.4	
N2b/N2c	25	59.5	11	40.7	
N3	6	14.3	14	51.9	
Supraclavicular node^f^	4	9.5	4	14.8	0.702
Extracapsular spread	18	42.9	23	85.2	<0.01**
Pathology					0.576
MD	11	26.2	10	37.0	
PD	27	64.3	14	51.9	
Undifferentiated	4	9.5	3	11.1	
Treatment					0.086
ND	3	7.1	2	7.4	
ND + adjuvant therapy	27	64.3	13	48.1	
RT alone	2	4.8	7	25.9	
Combined therapy	10	23.8	5	18.5	
Distant metastasis	7	16.7	12	44.4	0.012*

t test.

^f^Fisher’s exact test. Chi-Square test.

*p< 0.05,

**p< 0.01

**Fig 2:**
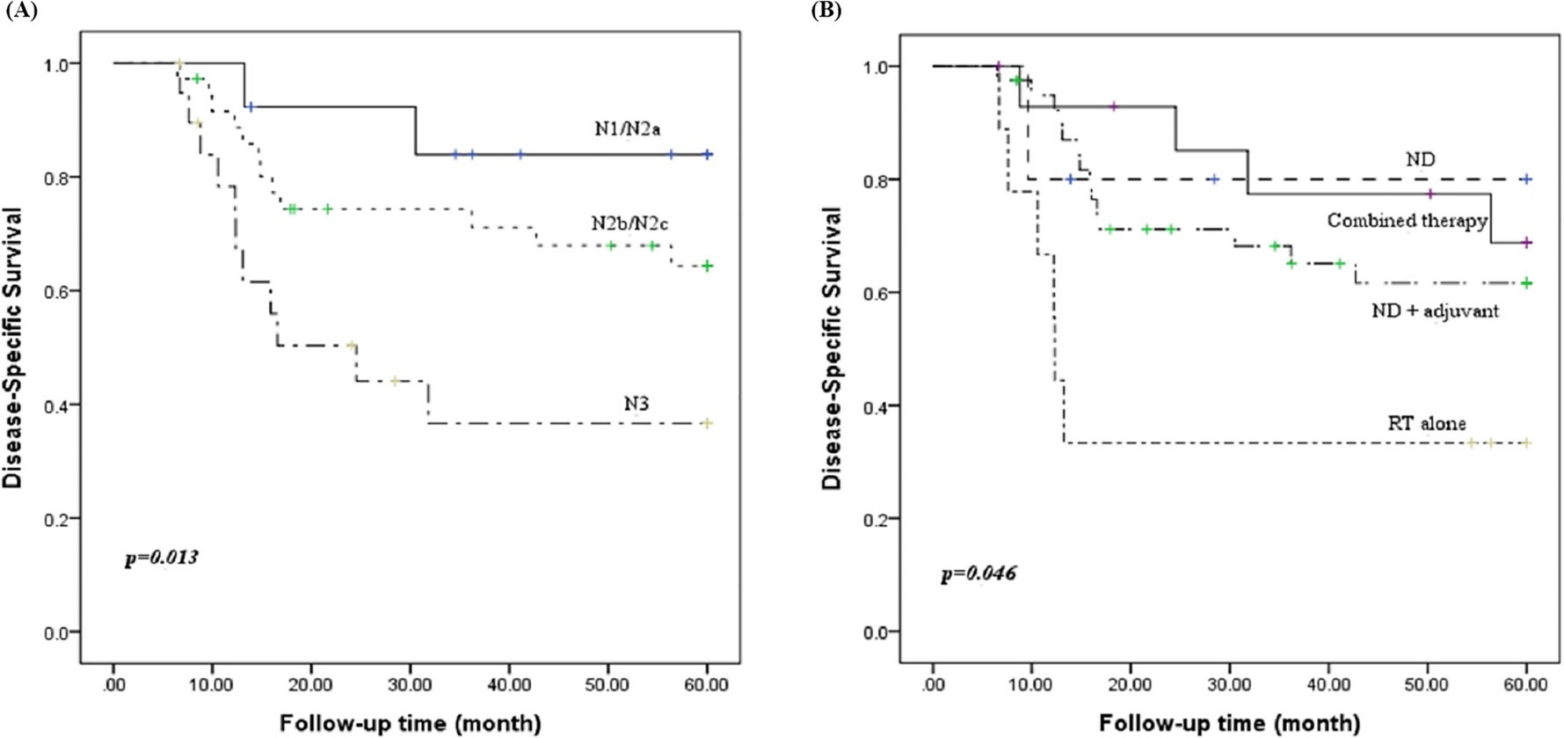
Kaplan–Meier survival curve of disease-specific survival according to the N stage (A) and treatment modalities (B).

The 5-year cumulative DSS rate of all these patients was 60.3%. The 5-year cumulative DSS rates of N1/N2a, N2b/N2c, and N3 patients were 83.9%, 64.3%, and 36.7%, respectively(p = 0.013) "Fig 2A". The 5-year cumulative DSS rates of ND, ND plus adjuvant therapy, RT alone, and combined therapy were 80.0%, 61.7%, 33.3%, and 68.8%, respectively(p = 0.046) "Fig 2B". The differences in the individual categorical variables for DSS are shown in Table 3.

**Table 3:** Comparison of parameters based on the status of survival.

	Alive (n = 44)		Death (n = 25)		*p* value
	n	%	n	%	
Age (mean ± SD)	55.6 ± 10.4		55.8 ± 10.9		0.938
Gender^f^					1.000
Male	36	81.8	21	84.0	
Female	8	18.2	4	16.0	
Occupation					0.701
Blue	16	36.4	8	32.0	
White	18	40.9	9	36.0	
None	10	22.7	8	32.0	
Alcohol	17	38.6	17	68.0	0.019*
Betel nut	13	29.5	15	60.0	0.013*
Smoking	25	56.8	21	84.0	0.021*
Diabetic mellitus^f^	6	13.6	6	24.0	0.330
Hypertension	11	25.0	5	20.0	0.636
Laterality					0.334
L	21	47.7	15	60.0	
R	20	45.5	7	28.0	
Bil	3	6.8	3	12.0	
Nodal stage					0.019*
N1/N2a	11	25.0	2	8.0	
N2b/N2c	24	54.5	12	48.0	
N3	9	20.5	11	44.0	
Supraclavicular node^f^	2	4.5	6	24.0	0.023*
Extracapsular spread	23	52.3	18	72.0	0.109
Pathology					0.504
MD	13	29.5	8	32.0	
PD	26	59.1	15	60.0	
Undifferentiated	5	11.4	2	8.0	
Treatment					0.187
ND	4	9.1	1	4.0	
ND + adjuvant therapy	26	59.1	14	56.0	
RT alone	3	6.8	6	24.0	
Combined therapy	11	25.0	4	16.0	
Distant metastasis	7	15.9	12	48.0	<0.01**
Appearance of primary^f^	5	11.4	5	20.0	0.478

t test.

^f^Fisher’s exact test. Chi-Square test.

*p< 0.05,

**p< 0.01

The uni- and multi-variate Cox regression analysis revealed that N3 stage, extracapsular spread, distant metastasis and treatment modality were significantly associated with neck recurrence (Table 4). Neck recurrence, N3 stage, supraclavicular node involvement, distant metastasis, alcohol consumption, betel nut chewing, smoking, and treatment modality were correlated with disease-specific mortality through univariate regression analysis. Nonetheless, multivariate regression analysis showed that supraclavicular node involvement, neck recurrence, and distant metastasis were the most important prognosticators (Table 5).

**Table 4:** Uni- and multivariate analysis of factors associated with 2-year neck recurrence.

	Univariate			Multivariate		
	HR	95% CI	*p* value	HR	95% CI	*p* value
Extracapsular spread	5.11	1.76–14.82	<0.01**	3.79	1.24–11.53	0.019*
Distant metastasis	2.88	1.34–6.17	<0.01**	3.05	1.26–7.42	0.014*
N stage						
N1/N2a	reference			reference		
N2b/N2c	2.16	0.48–9.73	0.318	3.20	0.64–15.88	0.155
N3	7.45	1.68–32.96	<0.01**	7.66	1.61–36.37	0.010*
Treatment						
ND	0.32	0.07–1.55	0.157	0.29	0.05–1.56	0.149
ND + adjuvant therapy	0.25	0.10–0.62	<0.01**	0.24	0.08–0.67	< 0.01**
RT alone		reference			reference	
Combined therapy	0.25	0.08–0.81	0.020*	0.37	0.10–1.39	0.141

Cox regression.

*p<0.05,

**p<0.01

**Table 5:** Uni- and multivariate analysis of factors associated with 5-year disease-specific mortality.

	Univariate			Multivariate		
	HR	95% CI	*p* value	HR	95% CI	*p* value
Supraclavicular node	5.59	2.08–15.01	<0.01**	4.57	1.20–17.39	0.026*
Distant metastasis	4.56	2.00–10.38	<0.01**	2.85	1.04–7.81	0.042*
Neck recurrence	7.99	3.31–19.27	<0.01**	4.21	1.37–12.91	0.012*
Alcohol	2.60	1.12–6.03	0.026*	2.24	0.71–7.03	0.168
Betel nut	2.41	1.08–5.36	0.032*	0.92	0.30–2.85	0.880
Smoking	3.41	1.17–9.95	0.025*	1.77	0.53–5.98	0.357
Nodal Stage						
N1/N2a	reference				reference	
N2b/N2c	2.40	0.54–10.74	0.251	2.26	0.32–15.98	0.416
N3	5.84	1.29–26.43	0.022*	3.84	0.55–26.90	0.175
Treatment						
ND	0.22	0.03–1.79	0.155	0.30	0.03–3.27	0.321
ND + adjuvant therapy	0.33	0.13–0.86	0.024*	0.27	0.07–0.97	0.044*
RT alone		reference			reference	
Combined therapy	0.23	0.06–0.82	0.023*	0.28	0.05–1.47	0.133

Cox regression.

*p<0.05,

**p<0.01

## Discussion

Cervical lymph node metastasis without diagnosis of a primary tumor was clearly defined as carcinoma of unknown primary by Comess et al. in 1957 [9]. The probable pathophysiology of this disease includes spontaneous regression, immune-modulated destruction of the primary cancer, faster growth rate of nodal metastasis, or sloughing of the necrotic cancer [10,11].

A review of literature revealed that the most frequent histopathological diagnoses of unknown primary cancers are SCC (65%-76%), followed by undifferentiated carcinoma, adenocarcinoma, and lymphoepithelial carcinoma [12]. The average age at diagnosis ranged from 55 to 65 years, with a male gender predominance. Level II was the most frequently involved nodal area of unknown primary SCCHN, followed by level III, with the N2 stages having apparent prevalence [3–5]. The level of the involved lymph nodes may offer insight into the location of the primary site and may also guide the diagnostic evaluation [13]. Unilateral lymph node involvement was more common, while bilateral lymphadenopathy was present in approximately 10% of the patients. In this study, the results are in line with most previous observations.

To date, the optimal therapeutic options for an unknown primary SCCHN remain ambiguous. For limited disease (N1, N2a, without ECS), some authors have recommended surgery as the mainstay for management owing to the acceptable clinical outcomes and side effects [6,14], while others have reported compatible results using definitive radiation [15]. Implementation of comprehensive neck dissection rather than elective neck dissection has been widely recommended, since a predictable pattern of nodal metastasis does not exist with unknown primary SCCHN. Combined therapy is mandatory for those in the advanced stages of disease, with the choice of divergent treatment strategies dependent upon the physician’s judgment and the patient’s performance status. Guntinas-Lichius et al. [4] have theorized that additional chemotherapy does not provide any survival benefits, however, the European Organization for Research and Treatment of Cancer (EORTC) demonstrated concomitant chemoradiation used as an adjuvant therapy confers a 13% improvement in locoregional control, a 25% risk reduction in disease progression, and a 30% risk reduction for death at five years [15]. The Radiation Therapy Oncology Group (RTOG) reported results showing that concurrent postoperative chemotherapy and radiotherapy significantly improved the rates of loco-regional control and disease-free survival [16]. In the present study, 5 out of 13 patients in the N1/N2a stage and 9 out of 56 patients in the advanced stage received a single treatment modality; either neck dissection or radiation alone. Two cases in the limited disease stage and 7 cases in the advanced stage had neck recurrence. Only 3 cases survived for more than five years among these fourteen patients. The control rate of patients receiving RT alone was very low. This might be due to lack of systematic effects of RT in advanced non- human papilloma virus (HPV) squamous cell carcinoma. Another possible explanation could be selection of patients with advanced disease deemed to be unresectable. ND plus adjuvant therapy and combined therapy had significantly higher LRC rates and DSS rates compared with those of the other treatment modalities “Figs 1B and 2B”. This finding hints that the single treatment model is inadequate for those with advanced stage disease.

More than half of the patients with locally advanced SCCHN developed loco-regional or distant relapses, which were usually detected within the first 2 years of treatment [1]. The loco-regional recurrence rate and distant metastasis rate after curative intent remedy, as found in the literature, varied from 9% to 59% and 3.5% to 38%, respectively [3,5,6,14]. These wide ranges were likely related to the disparate therapeutic modalities and selection of patients. The risk factors for loco-regional failure that were mentioned included nodal status, ECS, pathological subtype, treatment modality, positive margins, and male gender. ECS was generally diagnosed by histopathology, but could also be accurately predicted through CT scan or MRI findings [17,18]. In our series, 25 (44.6%) advanced stage cases involved loco-regional recurrence or persistent disease. N3 stage (HR 7.66, 95% CI 1.61–36.37, p = 0.010) and ECS (HR 3.79, 95% CI 1.24–11.53, p = 0.019) were the major factors which influenced neck control (Table 4). Twelve out of 27 cases that experienced loco-regional failure developed distant metastasis simultaneously or subsequently in this study. The incidence of distant metastasis in patients with loco-regional failure was higher than in those without loco-regional failure (44.4% versus 16.7%, p = 0.025). Therefore, the presence of loco-regional failure was strongly correlated with distant metastasis synchronously or metachronously (HR 3.05, 95% CI 1.26–7.42, p = 0.014). Consequently, the treatment strategy in our departments gave preference to surgery and postoperative adjuvant therapy whenever feasible.

Distant metastases were discovered in 5% to 40% of patients, usually within 2 years of treatment, and in correlation with the stage of disease at presentation [3]. Once distant metastasis in SCCHN was disclosed during follow-up, the median time to death was around 4 months [6]. The most common site of distant metastasis was the lung, followed by bone or liver. In this cohort study, 17 out of 19 distant metastases were detected within 2 years of treatment, with up to 14 cases having advanced stage with ECS. The mean time from first encounter to death was 6.1 months. The most prevalent sites of distant metastasis were in accordance with most studies.

The rate of emerging primary tumor varied from 7% to 20%, which was equivalent to the development of second cancers in the UADT or lungs [6,13]. The nasopharynx, tonsil and base of tongue were the most predominant sites mentioned in the literature. Although later development of a primary has been chronicled, the primary tumor typically emerged within 2 years after treatment and was associated with a poor prognosis [4]. Our data were generally compatible with most previous observations except for the most common site, whichwas oral cavity. This could be explained by the high rates of alcohol consumption, areca quid chewing and habitual smoking in our population. This was very different from occult primary tumors of head and neck in Western countries, which were typically HPV-related and were most likely originatedfrom the oropharynx. The prognosis of occult primary tumors of head and neck in Western countries was very different when compared to that of our patients. Thereported HPV prevalence for unknown primary SCCHN differed widely inprevious studies, between 22 and 74%[19,20]. The extent of HPV involvement in unknown primary SCCHN in Taiwan was currently unclear. However, the incidence of HPV-positivesquamous cell carcinoma of tonsilin Taiwan was reported to be only 12.6%[21].

The 5-year DSS rate of curative intent varied from 20% to 74% [2,3,5,14]. A rate of 60.4% was found in the present study. Several prognostic factors in cases of unknown primary cancers have been postulated, although a consensus has yet to be established. For example, some authors have found no significant effect of gender on survival rate, while others have shown a higher survival rate among females [5]. Iganej et al. [6] stated that an advanced nodal stage, along with the presence of ECS were significant adverse factors for survival. Both Braud et al. [2] and Guntinas-Lichius et al. [4] demonstrated that later development of a primary cancer was associated with a poorer survival rate. Our data show that none of the investigated parameters including age, gender, occupation, comorbidity, laterality, ECS, histopathology parameters, or the appearance of a primary tumorwere correlated with survival (Table 3). However, some prognostic parameters were noted; including neck recurrence, N3 stage, supraclavicular node involvement, distant metastasis, and unhealthy social habits.

Comorbidity with cardiovascular disease, respiratory disease, gastrointestinal disease, and diabetes impacted overall survival of the patients diagnosed with head and neck squamous cell carcinoma [22]. In this series, there was no significant correlation between disease-specific mortality rate and the rates of diabetic mellitus, hypertension among the subgroups of patients. The probable explanation for this is that we did not consider the severity of the comorbidities.

Neck recurrence, N3 stage, and distant metastasis were the key prognostic factors, which is mostly consistent with other head and neck cancers. Supraclavicular node involvement may hint that the origin may be the subclavicular lesion, which would indicate a poorer survival rate [13]. A possible interpretation is that the thoracic duct originates from the confluence of the cisterna chyli just below the diaphragm. The right lymphatic duct is responsible for lymphatic drainage of the right upper part of the trunk, and blockage of the lymphatic flow into the lower cervical area may allow for retrograde spread. In this series, eight cases had advanced stage with supraclavicular node involvement, but only three of the patients survived for longer than five years (HR 4.57, 95% CI 1.20–17.39, p = 0.026).

Poor lifestyle habits including excessive alcohol consumption, areca quid chewing, and tobacco smoking have been identified as the major risk factors for head and neck cancer. Patients with unknown primary cancer who smoked and consumed alcohol had a dismal survival rate [4,23]. Whether areca quid chewing worsens the survival rate of unknown primary cancer patients is unclear. Our statistics show that these three lifestyle habits could influence a patient’s survival, but they are less important when considered along with other parameters. Neck recurrence, distant metastasis, and supraclavicular node involvement appear to have a far greater impact on survival rates than alcohol consumption, betel nut chewing and smoking. To the best of our knowledge, this is the first study to analyze the relationship between betel nut chewing and survival in patients with an unknown primary SCCHN, and to further demonstrate that this lifestyle habit has a deleterious effect on survival.

There were several limitations in this study, including the relatively small number of patients, the retrospective and non-randomized study design, and the difficulty of isolating the effects of co-factors in statistical models, which have their own sets of limitations. Further research is warranted to quantify the effects of undesirable lifestyle habits and their interaction, to determine the severity of comorbidities, and to precisely delineate the occupational categories in unknown primary SCCHN.

## Conclusions

We conclude that composite therapy is mandatory for advanced unknown primary SCCHN. Supraclavicular node involvement and unhealthy lifestyle habits, such as betel nut chewing, indicate a poor prognosis.
